# Arsenic Methyltransferase and Methylation of Inorganic Arsenic

**DOI:** 10.3390/biom10091351

**Published:** 2020-09-22

**Authors:** Nirmal K. Roy, Anthony Murphy, Max Costa

**Affiliations:** Department of Environmental Medicine, NYU School of Medicine, 341 East 25th Street, New York, NY 10010, USA; Nirmal.Roy@nyulangone.org (N.K.R.); Anthony.Murphy@nyulangone.org (A.M.)

**Keywords:** metals, carcinogens, biotransformation

## Abstract

Arsenic occurs naturally in the environment, and exists predominantly as inorganic arsenite (As (III) and arsenate As (V)). Arsenic contamination of drinking water has long been recognized as a major global health concern. Arsenic exposure causes changes in skin color and lesions, and more severe health conditions such as black foot disease as well as various cancers originating in the lungs, skin, and bladder. In order to efficiently metabolize and excrete arsenic, it is methylated to monomethylarsonic and dimethylarsinic acid. One single enzyme, arsenic methyltransferase (AS3MT) is responsible for generating both metabolites. AS3MT has been purified from several mammalian and nonmammalian species, and its mRNA sequences were determined from amino acid sequences. With the advent of genome technology, mRNA sequences of *AS3MT* have been predicted from many species throughout the animal kingdom. Horizontal gene transfer had been postulated for this gene through phylogenetic studies, which suggests the importance of this gene in appropriately handling arsenic exposures in various organisms. An altered ability to methylate arsenic is dependent on specific single nucleotide polymorphisms (SNPs) in AS3MT. Reduced AS3MT activity resulting in poor metabolism of iAs has been shown to reduce expression of the tumor suppressor gene, *p16*, which is a potential pathway in arsenic carcinogenesis. Arsenic is also known to induce oxidative stress in cells. However, the presence of antioxidant response elements (AREs) in the promoter sequences of *AS3MT* in several species does not correlate with the ability to methylate arsenic. ARE elements are known to bind NRF2 and induce antioxidant enzymes to combat oxidative stress. NRF2 may be partly responsible for the biotransformation of iAs and the generation of methylated arsenic species via AS3MT. In this article, arsenic metabolism, excretion, and toxicity, a discussion of the *AS3MT* gene and its evolutionary history, and DNA methylation resulting from arsenic exposure have been reviewed.

## 1. Introduction

Arsenic is a metalloid found naturally in rocks and soil and is one of the major world-wide contaminants of drinking water. Arsenic has also been used as a wood preservative, pesticide, and also as a chemotherapeutic agent to treat promyelocytic leukemia [1]. Arsenic presence in various rocks and sediments is likely a result of weathered igneous rocks derived from volcanic exhalations and hot springs [2]. Arsenic concentrations in volcanic rocks were determined to be 3.5 ppm, whereas granites from Minnesota measured 1.0 ppm arsenic, on average. Arsenic in combination with elements including oxygen, chlorine, and sulfur are commonly referred to as inorganic arsenic (iAs), and they are the predominant form of environmental arsenic [3]. Arsenic is anthropogenically released into the environment due to coal burning, mining, and automobile emissions [3]. Arsenic was also used in industrial settings, primarily as a wood preservative in the form of copper chromated arsenate (CCA), commonly referred to as “pressure-treated” wood [3]. CCA was phased out of production in the United States in 2003; however, CCA-containing wood remains in use [3]. Tobacco also contains arsenic, which is absorbed from contaminated soil or soil containing naturally-derived traces of arsenic [3]. In addition, tobacco growers use pesticides containing arsenic [4]. Due to its presence in naturally- and anthropogenically-derived sources and its physiochemical properties, arsenic is capable of entering both surface and ground waters and, eventually, drinking water sources. Estimates of arsenic concentrations in rivers and lakes were determined to be as high as 1100 ppm [5]. Furthermore, agricultural products may contain arsenic due to the use of contaminated water in irrigation processes [6]. Altogether, there are many exposure pathways for arsenic, which is a particular problem considering the health effects that often accompany exposure.

Acute arsenic poisoning has been associated with abdominal pain, vomiting, diarrhea, and nausea [7]. Chronic oral exposure to arsenic causes the formation of darkened patches and small skin lesions [3]. From epidemiological studies, arsenic exposure has been shown to cause black foot disease, which is a unique peripheral vascular disease originally identified in the endemic area along the southwest coast of Taiwan [8,9]. In fact, individuals with higher arsenic exposure and lower capacity to metabolize arsenic were found to have a higher risk of developing black foot disease [8]. Moreover, epidemiological evidence shows that arsenic exposure through inhalation or drinking water causes lung, skin, and urinary bladder cancers and, as such, arsenic is classified as a Group I carcinogen by the World Health Organization (WHO) [10]. However, arsenic is not a classical carcinogen because it does not form DNA adducts or cause mutations in DNA except at unrealistically high doses, but it does induce mitotic arrest, genomic instability, and chromosomal aberrations [11,12]. Arsenic-induced carcinogenesis has also been suggested to occur by epigenetic mechanisms [13]. In addition, arsenic partially exerts its toxicity by inactivating over 200 enzymes, predominantly involved in DNA metabolism and repair [7]. Based on both human exposure potential and associated health risks, the WHO established a drinking water standard of 10 μg/L [4].

Exposure to arsenic in drinking water represents the greatest exposure pathway in Chile, Argentina, China, Mexico, Bangladesh, and the United States [4]. The first major incident of endemic disease caused by arsenic in drinking water was reported in the 1920s in Cordoba Province of Argentina [14]. In Taiwan, exposure to arsenic from drinking water was discovered in the late 1950s-early 1960s [9]. In India, arsenic poisoning was documented for the first time in 1983 when a patient presented with severe skin lesions [15]. Worldwide, as many as 140 million people are thought to be exposed to high levels of arsenic in drinking water [16]. Arsenic contamination of groundwater is a particular problem in Bangladesh and has been referred to as the largest mass poisoning of a population [15]. Out of 125 million people, between 35 and 77 million are at risk of consuming water with arsenic exceeding 10 μg/L [15,17]. In comparison, it has been estimated that 0.5–2.0, 2.0, and >3.0 million people are exposed to >10 μg/L arsenic in drinking water in Argentina, China, and in Vietnam and the United States, respectively [17]. Levels of arsenic in drinking water in Bangladesh, however, often exceed 50 μg/L, and maximum detected levels reaching 1000 μg/L and higher have been documented [18,19]. Similarly, a community of 6000 residents in the Andes Mountains in Argentina had arsenic contamination in their drinking water at levels as high as 290 μg/L [20].

In order to overcome arsenic exposure, humans have developed metabolic processes that allow for efficient excretion of arsenic in order to avoid high body burdens and systemic toxicity. The particular mechanism of arsenic metabolism and final chemical speciation of arsenic following metabolism are important considerations for its cumulative toxicity.

## 2. Arsenic Metabolism, Excretion, and Toxicity

Arsenic is one of the few metalloids that is metabolized in vivo. In humans and rodents, ingested arsenic compounds are detoxified and excreted following methylation. Detoxification and biotransformation pathways for iAs have only been proposed via methylated derivatives, first by the formation of monomethylarsonic acid (MMA) and then dimethylarsinic acid (DMA) [21]. Arsenic is not always fully methylated, and a portion of it is excreted as iAs and MMA. Arsenic methyltransferase is the enzyme responsible for metabolism and biotransformation of inorganic arsenic, and s-adenosylmethionine (SAM) is used as the methyl group donor [22].

Biotransformation to a methylated arsenic species to facilitate the removal of arsenic from the body was originally discovered in experiments with fungi [23]. In murine models, however, arsenic exposure via drinking water was successfully shown to produce elevated levels of methylated arsenic in urine [24]. Animals differ in the ratios of methylated arsenic species produced, and in their ability to methylate iAs [25]. Chimpanzee and marmosets do not methylate iAs, whereas hamsters and rabbits are enzymatically capable of converting iAs to DMA. Dogs and mice excrete approximately 81% and 71% DMA, respectively. Primary rat hepatocytes have been shown to methylate arsenic better than primary human cell lines, e.g., hepatocytes and keratinocytes [26], which has been suggested as one of the reasons why arsenic is less hazardous to animals. Elaborate studies on urinary excretion of arsenic metabolites were performed after repeated ingestion of iAs in humans. Upon single ingestion of iAs, 75% was shown to be excreted as methylated iAs, of which one-third was MMA and two-thirds DMA [27]. In order to develop an exposure history in industrial workers, the same authors also analyzed the excretion of arsenic metabolites in subjects after the ingestion of arsenic in graded doses for several days. About 60% of the ingested arsenic was excreted every day [28], and the composition of arsenic species excreted was similar to previous analyses (vide supra). Additionally, Vahter (1999) showed that the metabolic byproducts of arsenic are produced in different ratios in humans compared to most mammals and that humans excrete more MMA [25].

Although the mechanisms for the more rapid excretion of methylated arsenic compared to inorganic arsenic is not usually discussed, we hypothesize that this is due to the degree of protein-bound arsenic [29]. The same principle applies to the half-life of most drugs with those that are tightly protein bound having longer half-lives than those loosely bound. Thus, arsenite which is the major species in vivo, would have three protein binding sites which makes it more difficult to dissociate from a protein during passage through the kidney. In contrast, MMA and DMA would only have two and one protein binding sites, respectively, and would dissociate from protein more rapidly with a lower number of binding sites when passing through the kidney.

MMA is often considered more toxic than DMA, and higher body burdens of MMA have been suggested to be the reason why humans show toxic effects such as skin pigmentation and certain cancers [21]. However, complete methylation to the ultimate form As species is likely the most favorable outcome, despite the highly reactive and toxic intermediate metabolites. Yokohira et al. (2010) showed that more severe lesions were observed in urinary bladder epithelial cells in *AS3MT* knockout mice compared to wild-type mice [30]. In addition, severe systemic toxicity and urinary bladder cytotoxicity and regenerative hyperplasia were induced in *AS3MT* knockout mice [30]. Mechanistically, MMA^III^ and DMA^III^ were both shown to be more toxic and reactive compared to iAs^III^, and were shown to cause apoptosis via oxidative stress accompanied by loss of mitochondrial membrane potential and release of cytochrome C [31,32]. Therefore, it is likely that mammals have evolved to metabolize inorganic arsenic to its relatively non-cytotoxic pentavalent forms, but encounter high cytotoxicity due to intermediate metabolites, namely MMA^III^ and DMA^III^.

The cytotoxicity and genotoxicity of inorganic arsenic and its methylated species including methyloxoarsine (CH_3_As^III^O), iododimethylarsine (CH_3_As^III^I), monomethylarsonic acid (MMA^V^), dimethylarsinic acid (DMA^V^), monomethylarsonous acid (MMA^III^), and dimethylarsinous acid (DMA^III^) differ significantly [33]. For instance, iAs^III^ and iAs^V^ produced concentration-related linear increases in DNA damage as assessed by the single-cell gel assay (i.e., comet assay) using human peripheral blood lymphocytes, but were not significantly different from each other [33]. MMA^III^ and DMA^III^, on the other hand, were reported to be 54 and 77 times more potent than iAs^V^ or iAs^III^, respectively, and DMA^III^ was 270 and 386 times more potent than iAs^V^ or iAs^III^, respectively [33]. MMA^V^ and DMA^V^ were inactive and unable to damage DNA at high concentrations, however. Neither iAs^III^, iAs^V^, nor methylated pentavalent arsenic species produced significant nicking, strand breaks, or alkali labile lesions in DNA as assessed by either DNA nick assay compared to methylated trivalent As species [33]. While both trivalent methylated species MMA^III^ and DMA^III^ did show DNA damage in the DNA nick assay, this only occurred at abnormally high concentrations [33]. In agreement with other studies, indirect genotoxicity has frequently been observed with arsenic species and is more likely to occur than direct interaction with DNA at realistic concentrations [12,32,33,34]. Therefore, direct genotoxicity and mutagenesis resulting from arsenic species only occur at high concentrations, as exemplified by Klein et al. (2007), and an indirect mode of genotoxicity resulting from exposure to arsenic is more realistic [12].

Moe et al. (2016) compared the cytotoxicities of iAs^III^ and iAs^V^ in human urinary bladder carcinoma T24 cells and human lung adenocarcinoma A549 cells, and iAs^III^ was reported to have a significantly lower IC_50_ than IAs^V^, which represents the concentration of arsenic species that results in a 50% reduction in cell index compared to unexposed cells and reflects cell death [21]. Moreover, the cytotoxicities of 14 additional arsenic species were determined. The reported cytotoxicities of the arsenic species tested were as follows: phenylarsine oxide, PAO^III^; >methylarsine oxide, MAO^III^; >MMA^III^; >DMA^III^; >dimethylarsinic glutathione, DMAG^III^; >dimethylmonothioarsinate, DMMTA^V^; >As^III^; > monomethyltrithioarsonate, MMTTA^V^; >As^V^; >dimethyldithioarsinate, DMDTA^V^; >DMA^V^; >MMA^V^; >roxarsone, Rox; >and p-arsanilic acid, >p-ASA [21]. Notably, all trivalent As species were the most cytotoxic in both cell lines tested, with the exception of DMMTA^V^. Rox, a common pesticide used in the poultry industry, and p-ASA were the least cytotoxic as their IC_50_ values exceeded 9 and 6 mM in A549 and T24 cells, respectively [21,35]. The carcinogenicity of methylated As species was also tested in liver and prostate cell lines, and MMA^III^ was determined to cause an increase in invasiveness and colony formation in soft agar—a measure of anchorage-independent growth and hallmark of cancer [34]. While there may be slight discrepancies in the reported cytotoxicities between MMA^III^ and DMA^III^, inorganic and organic trivalent arsenic species are certainly more cytotoxic compared to pentavalent forms [21].

## 3. Discovery of the *AS3MT* Gene and Protein

In order to identify the mRNA that codes for the enzyme responsible for arsenic metabolism, Lin et al., (2002) purified the protein from rat liver cytosol using chromatofocusing and affinity chromatography [36]. Purified protein was then electrophoresed on SDS-polyacrylamide gel, stained with Coomassie blue, extracted from the gel, and subjected to trypsin digestion. They sequenced the small hydrolyzed peptides and designed degenerate oligonucleotide primers from the peptide sequences to amplify the mRNA with PCR. The cDNA sequence for *AS3MT* was deduced from the peptide consisting of 369 amino acids with a molecular weight of 41Kd. Since then, many AS3MTs have been identified throughout the animal kingdom and in lower organisms. Peptide lengths (amino acids; aa) and molecular weights (kDa) of AS3MTs in animals are as follows: 375 and 41.8 for humans [37], 375 and 41.7 for rhesus monkeys [38], 376 and 41.8 for mice [39], and 382 and 41.9 for chickens, respectively (GenBank accession No. XP_421735).

In order to isolate and purify the enzyme responsible for methylation of arsenic, radioactive SAM was utilized to assay enzymatic activity [40]. Researchers purified two methyltransferases for arsenic and MMA separately from rabbit livers. When electrophoresed on an SDS polyacrylamide gel, both of the purified proteins migrated together, suggesting that a single enzyme responsible for the methylation of iAs to MMA and eventually to DMA. The molecular weight for AS3MT was 60 kDa. The difference in molecular weights between rabbit enzymes and enzymes from other species has been discussed in several articles [41,42]. In 2016, automated sequencing confirmed that the rabbit peptide sequence (GenBank accession number XM_008270429) was more than 90% similar to other known AS3MT enzymes (discussed later) with a predicted molecular weight of 41.6 kd. Zakharyan et al. (1999) also were able to purify AS3MT from human hepatocytes to establish that this enzyme is also present in humans [43].

Apart from studies on the influence of genetic polymorphism and AS3MT enzyme activity, extensive analysis of the structure and enzymatic activity was carried out [44]. Two *AS3MT* genes were identified in a eukaryotic alga, *Cyanidioschyzon* sp., and were shown to confer arsenic resistance in *Escherichia coli* and methylate iAs [45]. Several cysteine residues (72, 174, and 224) were identified as necessary for the methylation of iAs [46]. To investigate their properties, they were substituted with Alanine to maintain the neutrality of the new amino acid. Mutants at residue 72 can still methylate MMA to DMA, but residues 174 and 224 were essential for both steps of methylation. Crystallographic studies of this algal enzyme indicated that incubation with SAM orients the binding domain such that SAM is in close proximity with iAs bound to the enzyme for the methylation reaction to proceed [47].

The contribution of cysteine residues to methylation ability by human AS3MT were investigated individually [48]. Important cysteine residues (72, 174, and 224) in the algal species now line up with human 61, 156, and 206 residues. Additionally, the cysteine residue at 32 was also determined to be important in the methylation reaction [48]. When the AS3MT protein sequences were aligned, cysteines at positions 32, 61, 156, and 206 were conserved in primates, as depicted by aligning the AS3MT peptide sequences [49]. Attempts were made to purify the enzyme in vitro, but it was reportedly difficult to produce it in large quantities for crystallographic studies [50]. Changes were made in the coding sequences, such that this cDNA can be utilized to synthesize AS3MT in large quantities in vitro for biochemical studies [48]. Similar to algal enzymes, mutations at any of the four positions abolished the activity of the enzyme. Mutants with residue at 32 and 61 can still methylate MMA to DMA, but, mutants at 174 and 224 cannot take part in any of the two methylation steps [48]. With the discovery of new polymorphic sites in human AS3MTs [51], all eight mutant proteins showed decreased methylation activity in vitro due to among other factors, their low substrate affinity, and stability [52].

### 3.1. Analysis of AS3MT Genomic Sequences

During evolution, select species likely required different handling of iAs to survive, and as a result, they developed the ability to methylate and excrete different forms of organic arsenic. This differential evolution might have left a signature in the way the coding sequences are arranged in the genome. Attempts were made to investigate whether species with a comparable ability to methylate iAs evolved similarly and exhibited a signature in how their exons were arranged.

**Figure 1:**
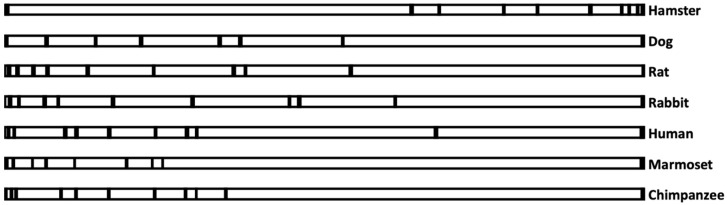
**Exon–intron arrangements of seven coding sequences in the *AS3MT* genome.** Exons containing only the coding sequences were indicated according to the way they are arranged in terms of distance from each other. Dark small rectangles represent the exons, whereas the large open rectangle represents introns in the genome of individual species from the start of translation to the stop codon. Space between the two dark rectangles represents the length between two exons. Genbank accession number for the sequences are NW_020822501 (hamster), NC_006610 (dog), CM000231 (rat), NC_013686 (Rabbit), AC009144 (human), NC_013907 (marmoset), and NBAG03000216 (chimpanzee).

Figure 1 shows a similarity among the species that have been studied. For example, in hamsters, exons in the *AS3MT* gene were predominantly near the 3′ end, whereas exons in chimpanzees, humans, and marmosets were mostly near the 5′ end of the gene. In other species, exons were mostly distributed throughout the gene. Surprisingly, in both marmosets and chimpanzees, which cannot methylate iAs, their exons were also arranged entirely at the 5′ end of the gene. This could be entirely by chance, or at some point during evolution, such configuration was needed to handle iAs in a similar fashion for survival. Species differences should be further investigated to understand the significance of the distribution of exons in the 5′ end of the gene.

On the other hand, rabbits and hamsters excrete about 50% arsenic as DMA, but their exon–intron arrangements were different from each other (exons are in the 3′ end of the gene in hamsters and mostly in the 5′ end of the gene in rabbits). It seems that whenever species have some ability to metabolize iAs, their exon–intron arrangements are random. Perhaps these species have evolved in entirely different situations or environments to metabolize iAs in differently.

### 3.2. The Relationship between AS3MT Promoter Sequences and the Ability to Methylate Arsenic

Exposure to iAs results in the formation of superoxide oxygen radicals and hydrogen peroxide, yet the exact pathway for the generation of these reactive species is not well studied. In the brain, however, arsenic was shown to mediate the production of reactive oxygen species (ROS) [53]. Experiments were performed in which sodium arsenite was directly infused into the brain of rats. This caused a significant increase in the formation of oxygen radicals [53]. A pathway for the generation of ROS was predicted; As(III) in presence of oxygen was converted to As(V) and generated hydrogen peroxide [53].

Cells tend to express antioxidant genes under a state of enhanced oxidative stress [54]. One example of these genes is *NRF2*, which binds to antioxidant response elements (AREs) in promoter regions to regulate the expression of various enzymes to quench oxidative stress [55,56]. The putative ARE was identified by mutational analysis, and the essential functional core sequence was shown to be RTGACnnnGC [57]. NRF2 was found to be regulated by the binding of an inhibitor known as KEAP1, which prevents the translocation of NRF2 into the nucleus. Specifically, NRF2 is regulated by protein stability as KEAP1 functions as an adaptor for CUL3-based E3 ligase to facilitate proteasomal degradation of NRF2 [55,58]. NRF2 is also regulated in a KEAP1-independent manner [59]. It would be interesting to investigate whether the presence of AREs in AS3MT correlates with the degree of arsenic methylation in different animal species and if ROS generation resulting from arsenic exposure is responsible for AS3MT induction, and whether this involves NRF2, given the presence of multiple AREs in the *AS3MT* promoter.

Genomic sequencing revealed that chimpanzees have the *AS3MT* gene, but cannot methylate iAs. Their genomic sequence contains one consensus and three 90% consensus AREs. The human promoter sequence is almost identical to the chimpanzee sequence and contains only one additional ARE with 90% similarity. However, in humans, more than 60% of urinary arsenic is DMA. Additionally, rats and rabbits have a similar number of AREs, and rabbits excrete 50% DMA compared to only 20% for rats. Therefore, based on this analysis it is unlikely that the presence or absence of AREs in the *AS3MT* promoter dictates the ability to methylate or not methylate arsenic. This suggests that other factors besides AREs, such as epigenetic changes, might be responsible for the differential metabolism of arsenic.

Despite the lack of evidence supporting a connection between the number of ARE binding sites in the *AS3MT* promoter, subsequent AS3MT induction, and ability to metabolize As, NRF2 induction via ROS is likely involved in AS3MT induction. If true, this would suggest that rather than differences in AS3MT induction dictating As metabolism, other factors potentially upstream of NRF2 or involving the antioxidant response system may be at play. Arsenic has been determined not to disrupt KEAP1/NRF2 association in the cytoplasm, and instead, alternative pathways of activation, such as by crosstalk with redox-sensitive transcription factors may be responsible for nuclear retention and localization of NRF2 [60,61,62,63]. One study, in particular, provides evidence that ROS production may impact AS3MT expression via NRF2. McNeil et al. (2015) developed a murine model with targeted deletion of Gch1, an enzyme required for BH4 synthesis and necessary for NO production via iNOS, yet permits for ROS production [64]. Upon deletion of Gch1 and indirect depletion of BH4, As3mt expression was found to be altered due to an impact on cellular redox status [64]. Ingenuity pathway analysis predicted significant modulation of the Nrf2 pathway, and a decrease in upstream Nrf2 activity in Gch1-null mice [64]. As3mt expression was concluded to be independent of iNOS, dependent on BH4 and Gch1, and altered by ROS or RNS. Stamatelos et al. (2013) provide insight into how this process might unfold with respect to perpetual redox reactions throughout the metabolism of iAs^V^ to the final methylated form of iAs, DMA^V^ [63]. In the proposed toxicodynamic/toxicokinetic model, the lifecycle of iAs is described that incorporates the cyclic induction of antioxidants, activation of Nrf2 via ROS, and inhibition/negative feedback interaction with GSH [63]. A direct connection between NRF2 activation following arsenic exposure and *AS3MT* induction via NRF2 has yet to be fully investigated, however.

### 3.3. Evolution of the AS3MT Gene

**Figure 2:**
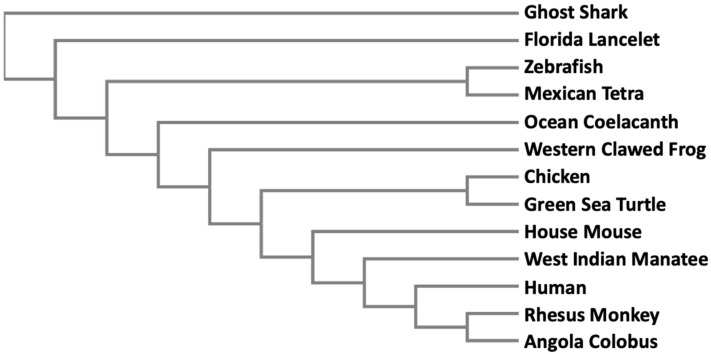
**Phylogenetic analyses of 13 full-length AS3MT peptide sequences in vertebrate species.** Simple phylogeny application from EMBL-EBI was used to generate a phylogenetic tree as described in the text. See text for Genbank accession numbers.

Since methylation is the only known way to biotransform and detoxify iAs in organisms, it is not surprising that the *AS3MT* gene is found in many different taxa extending from bacteria to higher mammals. In a simple phylogenetic tree, we have included a small set of vertebrate animals where *AS3MT* has been identified (Figure 2). Full-length peptide sequences were subjected to Clustal Omega alignment available from EMBL-EBI [65]. The resultant multiple sequence alignment was then entered into a phylogeny application from EMBL-EBI [66] to generate the phylogenetic tree. Peptide sequences were obtained from GenBank. The accession numbers of the sequences are: ghost shark (XP_007882715.1), Florida lancelet (XP_002609029.1), zebrafish (NP_001034928.1), Mexican tetra (XP_007253206.1), ocean coelacanth (XP_006007443.1), western clawed frog (NP_001135714.1), chicken (XP_421735.3), green sea turtle (XP_007057932.1), house mouse (NP_065602.2), West Indian manatee (XP_004370149.1), human (Q9HBK9), rhesus monkey (XP_001113391.2), and Angola colobus (XP_011812758.1). This phylogenetic tree clearly indicates that AS3MT proteins in the vertebrates belong to a single clade, and gradually evolved from fish to amphibians to higher mammals.

The evolution of the *AS3MT* gene has been investigated rather extensively [49]. Palmgren et al. (2017) examined 150 genes from 134 species. The genes from animals formed a single clade, proving that it might be advantageous for them to maintain this gene. The same authors also investigated three individual domains of the *AS3MT* genes. The central and the amino terminus domains are responsible for binding with arsenic and SAM, respectively. When subjected to phylogeny, these two regions formed a similar tree, indicating that enzymatic activity had been evolutionarily preserved.

Chimpanzees are known to possess *AS3MT* mRNA that codes for a 205 aa peptide and this truncated protein was assumed to be catalytically inactive [67]. Later, genome sequencing revealed that they also have three other isoforms of AS3MT, *Pan troglodytes* X1, X2, and X3 with GenBank accession numbers XP_009457416, XP_508007, and XP_016774748, respectively. When these sequences were aligned, all three necessary cysteines in the active site align with the human sequence [49]. The only residue that differed from the human sequence was glutamic acid at position 141. However, this residue is not conserved in other species that can methylate arsenic like *Chlamydomonas* [68]. Further investigation is necessary to determine differences in isoform activity in chimpanzees and any sequence dissimilarities in enzymes that can methylate arsenic.

## 4. DNA Methylation and Arsenic Exposure

Investigators have shown that arsenic exposure leads to changes in global methylation and methylation of specific promoters [69]. For example, low expression of tumor suppressor gene *p16* was documented in urothelial tumors from patients exposed to arsenic in Taiwan, and methylation-specific PCR showed that arsenite exposure induced *p16* gene hypermethylation in human uroepithelial cells [70]. *p16* expression was also found to be significantly associated with urothelial tumors from black foot disease areas compared to non-black foot disease areas [70]. Methylation of the *MLH1* promoter, an important gene in the mismatch repair pathway, was found in gastric and colorectal carcinoma [71]. *p16* and *MLH1* promoter methylation was investigated in a group of individuals exposed to elevated levels of arsenic in their drinking water [72]. Elevated arsenic concentrations in drinking water resulted in higher promoter methylation of both these genes. Increasing amounts of iAs in urine were found to be positively correlated with *p16* promoter methylation, whereas less toxic DMA was negatively correlated with *p16* promoter methylation. *p16* gene expression has also been shown to be negatively correlated with increasing amounts of iAs in urine. In addition, promoter methylation of *MLH1* was shown to be correlated with urinary arsenic concentrations.

Individuals with AS3MT haplotype I (16.5% of the above population) showed slower methylation of iAs, resulting in more arsenic and MMA and less DMA in their urine [73]. Subjects with one or two copies of the haplotype showed higher methylation of *p16* compared to null carriers [72]. *MLH1* methylation was not elevated in this population with this AS3MT haplotype [72]. Therefore, individuals with the slow metabolizing AS3MT were inadvertently exposed to higher amounts of iAs due to the lack of metabolism, which eventually contributed to reduced expression of tumor suppressor gene *p16*. This was proposed to be one of the pathways leading toward carcinogenesis by iAs [72]. There has also been speculation that because arsenic is methylated, significant depletion of cellular SAM occurs that results in global hypomethylation of DNA and histones.

## 5. Single Nucleotide Polymorphisms and AS3MT Activity

Arsenic methylation has been compared in two different populations exposed to iAs. One population was women exposed to arsenic in drinking water in the Argentinean Andes. They showed a lower percentage of highly toxic MMA and a higher percentage of DMA compared with most other populations [73]. The population in Argentina used in the study had been drinking arsenic-contaminated water for generations. Three polymorphic SNPs in the *AS3MT* gene significantly altered the ratios of MMA/DMA concentration in their urine. Wild-type homozygotes had 33% of MMA compared to variant homozygotes. It is plausible that the Argentinian population acquired this set of advantageous polymorphisms to efficiently metabolize arsenic because they were drinking arsenic-contaminated water for thousands of years [74].

Over the last 20 to 40 years, people in Bangladesh have been using tube wells to obtain their drinking water. Although urinary arsenic concentration was lower in the Bangladesh population compared to the Argentinian population, percentage-wise they have lower, less toxic DMA and higher iAs and MMA in their urine [73]. In the Bangladesh population, the advantageous polymorphisms were opposite compared to the Argentinian population. Individuals who were wild-type homozygotes showed a higher percentage of MMA and iAs compared to less toxic DMA and they show typical characteristics of arsenic toxicity. It is also feasible that people in Bangladesh (unlike the Argentinian population) did not have an opportunity to acquire the advantageous polymorphisms.

An advantageous group of people in the Argentinian population metabolize arsenic more efficiently compared to people in Bangladesh due to different SNPs. Since all of these SNPs are in the introns, the mechanism of this altered metabolic activity remains a mystery. To shed some light on this problem, promoter methylation of *AS3MT* and its surrounding genes and its impact on arsenic metabolism was investigated [75]. The *AS3MT* gene is located in the 10q24 region of the human chromosome with 10 exons and spans 32 kilobases [76]. When methylation at the CpG sites in *AS3MT* genomic DNA was examined in two populations, the Argentinian population showed significant methylation of a few sites compared to the Bangladeshi population. In line with the thought that more methylation might lower the expression of the gene, the Argentinian population showed reduced expression of AS3MT [75]. Increased expression of AS3MT is not necessarily an efficient and effective way to metabolize iAs. In fact, one particular polymorphic AS3MT (287The) was highly expressed in vitro, compared to the wild type, but the metabolic products of iAs were predominantly highly toxic MMA and less DMA [76]. This polymorphic locus occurs in the Argentinian population at a very low level, which is another reason why this population can metabolize iAs better. Further investigation is needed to understand why the lower expression of AS3MT can metabolize iAs to less toxic DMA, whereas higher expression of AS3MT yields more toxic MMA.

A similar phenomenon had been described in several different populations living in the Atacama Desert region (i.e., southern Peru, Bolivia, northern Chile, and Argentina) for thousands of years [77]. One population from Quebrada Valley of Chile, where arsenic levels in drinking water reache in excess of 200 μg/L, can metabolize arsenic efficiently and can rapidly reduce arsenic burden via urination [78]. Another population, namely, the Chinchorro settled in the Camarones valley in Chile with elevated levels of arsenic in natural water and showed increased rates of spontaneous abortions and childhood deaths, which prompted them to initiate mummification processes to cope with the loss [79]. This hypothesis is based on the finding that some archaeological sites had mummies of predominantly newborns and children [80]. When the arsenic content of bones and hair were analyzed, the arsenic burden was lower in the population 3000 to 500 years before present (BP) compared to the population 7000 to 3000 years BP, possibly because by this time they acquired advantageous polymorphisms in the *AS3MT* gene to efficiently metabolize arsenic [79].

## 6. Conclusions

Arsenic exposure is a common occurrence throughout the animal kingdom. Methylation has been utilized to detoxify arsenic and AS3MT plays a pivotal role in this process. Partially methylated product of iAs, MMA is generally considered more toxic than DMA. Trivalent arsenic species, namely MMA^III^ and DMA^III^, are more toxic compared to pentavalent species. Although many species seem to have this enzyme, some species cannot fully methylate arsenic. On the other hand, others can metabolize and excrete 70% to 80% arsenic in the form of DMA. Even in some human populations, increasing amounts of toxic MMA and lower amounts of DMA have been detected in their urine compared to different populations. Some advantageous SNPs and differential methylations have been argued to be the reason for this population difference. The difference in the excretion rate of arsenite, MMA, and DMA may also relate to protein binding. Arsenite binds very tightly to proteins and is not released during passage through the kidney, whereas MMA and DMA have reduced capacity to bind to proteins [29]. In addition, excretion rates are inversely proportional to the extent of protein binding. In conclusion, arsenic exposure and metabolism have been studied extensively for decades, yet much remains to be revealed in order to fully understand arsenic toxicity.
